# Dysregulation of miR-210 is involved in the development of diabetic retinopathy and serves a regulatory role in retinal vascular endothelial cell proliferation

**DOI:** 10.1186/s40001-020-00416-3

**Published:** 2020-05-29

**Authors:** Chengyu Yin, Xiangqiang Lin, Yafei Sun, Xinli Ji

**Affiliations:** Department of Ophthalmology, Qingdao Chengyang People’s Hospital, No. 600, Changcheng Road, Qingdao, Shandong 266000 China

**Keywords:** Diabetic retinopathy, MicroRNA-210, Diabetes mellitus, Diagnosis, Vascular endothelial cell, Proliferation

## Abstract

**Background:**

Diabetic retinopathy is a common complication of diabetes mellitus (DM). The purpose of this study was to investigate the expression and clinical significance of miR-210 in DR patients and explore the regulatory effect of miR-210 on vascular endothelial cell function under high-glucose condition.

**Methods:**

Quantitative real-time PCR was used to estimate miR-210 expression. A receiver operating characteristics curve (ROC) was plotted to evaluate the diagnostic value of miR-210. Human umbilical vein endothelial cells (HUVECs) were used and treated with high glucose (30 mM), and the cell proliferation was assessed by MTT assay.

**Results:**

Serum expression of miR-210 was upregulated in DR patients compared with DM without DR patients and healthy controls. The expression of miR-210 in proliferative DR (PDR) patients was higher than non-proliferative DR (NPDR) patients. The increased serum miR-210 could be used to distinguish DR cases from healthy individuals and also simple DM patients, and can screen PDR cases from NPDR cases. The overexpression of miR-210 promoted HUVEC proliferation, while the knockdown of miR-210 resulted in the opposite effect under a high-glucose condition.

**Conclusion:**

The data of this study demonstrated that serum increased miR-210 serves as a diagnostic biomarker in DR patients and may have the ability to predict DR development and severity. The regulatory effect of miR-210 on vascular endothelial cell proliferation under high-glucose condition, indicating its therapeutic potential in the treatment of diabetic vascular diseases.

## Background

Diabetic retinopathy (DR) is one of the most frequent microvascular complications of diabetes mellitus (DM) [1]. It is considered a leading cause of blindness in the working-age adult, leading to a huge economic and social burden [2]. The risk of losing vision is 25-fold in the population with diabetes than the people without diabetes [3]. The pathogenesis of DR is characterized by endothelial cell dysfunction, retinal neovascularization, the formation of acellular capillaries, microaneurysm formation and thickening of the basement membrane [4]. Accumulated studies demonstrated that the function of retinal vascular endothelial cells is firstly impaired in the development of DR, the cell proliferation is inhibited and the cell apoptosis is enhanced upon hyperglycemia condition [5]. The early-stage DR without neovascularization is defined as non-proliferative DR (NPDR), and the cases with neovascularization that attributed to the promoting effects of vascular endothelial growth factor and hypoxia-induced factor on retinal blood capillary belong to advanced DR and are defined as proliferative DR (PDR) [6]. Thus, the identification of molecules involving the regulation of vascular endothelial cell function and angiogenesis may help to improve the treatment of DR.

Abnormal microRNAs (miRNAs) have been identified in various human diseases with an important functional role in disease pathogenesis [7]. Numerous miRNAs have been demonstrated to be involved in the progression of DM, as well as various complications of DM, including DR [8, 9]. For example, miR-21 has been determined as a novel therapeutic target of DR by regulating retinal neovascularization and inflammation [10]. The knockdown of miR-218 might be a promising therapeutic approach of DR by promoting retinal pigment epithelium proliferation and inhibiting cell apoptosis [11]. Dysregulation of miR-210 is involved in the progression of DM [12], and its promoting effect on angiogenesis by increasing vascular endothelial cell proliferation has also been reported in several previous studies [13, 14]. However, the expression data of miR-210 in DR has not been investigated.

Considering the reported important role of miR-210 in DM and endothelial cell function, this study aimed to assess the expression patterns of miR-210 in DR patients, as well as its clinical significance in diagnosis and severity prediction. Furthermore, this study also investigated the regulatory effect of miR-210 on the cell proliferation of HUVECs upon high-glucose condition.

## Materials and methods

### Patients and clinical samples collection

This study was performed with the approval by the Ethics Committee of Qingdao Chengyang People’s Hospital, and all participants wrote the informed consent before sampling. A total of 150 type 2 DM patients and 60 age- and gender-matched healthy volunteers were recruited in this study from Qingdao Chengyang People’s Hospital between 2015 and 2017. The demographic characteristics of the two groups are recorded in Table 1. Exclusion criteria were as follows: patients accompanied by diabetic ketoacidosis, diabetic hyperosmolar coma and other acute complications of diabetes; patients having a history of myocardial infarction, coronary artery bypass surgery; patients with liver or renal dysfunction; patients suffering from peripheral vascular diseases and other endocrine and metabolic diseases; patients with cancer. All the participants received an ophthalmologic examination. The DM patients were diagnosed in line with the American Diabetes Association criteria [15] and divided into three groups, including 40 DM without DR patients (NDR group), 60 NPDR patients (NPDR group) and 50 PDR patients (PDR group), based on the fundus fluorescence angiography (FFA) results. The diagnosis of DR was determined by two experienced physicians following the designated staging criteria in the fundi disease academic conference [16]. The demographic and clinical data of the study population are recorded in Table 2.

**Table 1:** Comparison of the demographic between healthy controls and DR group

Variables	HC (n = 60)	DR (n = 150)	*P* value
Age (year)	50.85 ± 5.20	50.82 ± 3.85	0.969
Gender (male/female)	28/32	77/73	0.541
BMI (kg/m^2^)	21.81 ± 2.20	22.17 ± 2.68	0.315

Data are expressed as the mean ± SD or number. Gender distribution between the two groups were evaluated by using Chi-square test, while Student’s t test was used for other variables analysis

*HC* healthy control, *DR* diabetic retinopathy, *BMI* body mass index

**Table 2:** Comparison of the demographic and clinical data of patients in each group

Group	Age (year)	Gender (male/female)	BMI (kg/m^2^)	Disease course (year)	FPG (mmol/l)	TG (mmol/l)	TC (mmol/l)	HbA1c (%)	FINS (mIU/I)	HOMA-IR
HC	50.85±5.20	28/32	21.81±2.20	–	5.14 ± 0.83	1.47±0.17	4.28±0.49	4.92±0.46	5.38±0.54	1.29±0.08
NDR	50.23±3.81	23/17	21.78±1.84	4.27±0.72	6.15±0.58*	1.50±0.14	4.40±0.51	6.61±0.59*	5.41±0.65	1.57±0.10*
NPDR	50.73±3.80	28/32	22.07±2.73	9.55±1.57^#^	7.42±0.31*^,#^	1.52±0.28	4.50±0.75	7.31±0.28*^,#^	5.46±0.79	1.61±0.28*
PDR	51.40±3.93	26/24	22.61±3.12	12.68±1.43^#,&^	8.51±0.44*^,#,&^	1.55±0.26	4.57±0.73	8.00±0.34*^,#,&^	5.51±0.63	1.84±0.23*^,#,&^

Data are expressed as the mean ± SD or number. Multiple comparisons were made using ANOVA followed by Tamhane’s T2 tests, while the gender distribution among groups were evaluated by using Chi-square test

*HC* healthy control, *NDR* DM without DR patients, *NPDR* non-proliferative DR, *PDR* proliferative DR, *FPG* fasting plasma glucose, *TG* triacylglycerol, *TC* total cholesterol, *HbA1c* glycosylated hemoglobin, *FINS* fasting insulin, *HOMA-IR* homoeostasis model assessment of insulin resistance

*P < 0.05 compared with HC group; ^#^*P* < 0.05 compared with NDR group; ^&^*P* < 0.05 compared with NPDR group

Blood samples were collected from the participants, and serum samples were isolated from the blood by centrifugation and stored at −80 °C for further analysis. The clinical characteristics of the patients, including disease course, fasting plasma glucose (FPG), triacylglycerol (TG), total cholesterol (TC), glycosylated hemoglobin (HbA1c), fasting insulin (FINS) and the calculated Homoeostasis model assessment of insulin resistance (HOMA-IR), were recorded for further analysis.

### Cell culture and treatment

Human umbilical vein endothelial cells (HUVECs) were purchased from the Cell Bank in the Shanghai Institute for Biological Sciences of the Chinese Academy of Sciences (Shanghai, China) and cultured in RPMI-1640 medium (Gibco, Thermo Fisher Scientific, Inc., Waltham, MA, USA) containing 10% fetal bovine serum (FBS; Sigma-Aldrich, Merck KGaA, Germany) at 37 °C in a humidified incubator with 5% CO_2_. To obtain HUVECs under hyperglycemia condition, HUVECs were subjected to 30 mM glucose (HG group) as the previous study reported [17]. Besides, a glucose control group was performed by treating the cells with 5 mM glucose (LG group).

### Cell transfection

To regulate the expression of miR-210 in HUVECs, miR-210 mimic, miR-210 inhibitor and the negative controls (mimic NC and inhibitor NC) were separately transfected into the cells by Lipofectamine 3000 (Invitrogen, Carlsbad, CA, USA) following the manufacturer’s instruction. After 24 h of transfection, cells were received glucose treatment.

### RNA extraction and quantitative real-time PCR (qRT-PCR)

Total RNA in serum and cells was extracted using TRIzol reagent (Invitrogen, Carlsbad, CA, USA), and then reversed transcribed into cDNA by a PrimeScript RT reagent kit (TaKaRa, Shiga, Japan) based on the manufacturer’s protocol. The following qPCR was performed using a 7300 Real-Time PCR System (Applied Biosystems, USA) and the SYBR green I Master Mix kit (Invitrogen, Carlsbad, CA, USA). The relative expression of miR-210 was calculated using 2^−ΔΔCt^, and U6 was used as the internal control for miRNA quantification.

### MTT assay

HUVECs were seeded into 96-well plates with a cell density of 5 × 10^3^ cell/well and cultured at 37 °C for 78 h. At the culture time points of 0, 24, 48 and 72 h, 10 μL MTT solution (25 mg/mL; Sigma-Aldrich, Merck KGaA, Germany) was added to the cells with further 4 h of incubation. After removing the medium, 150 μL DMSO (Sigma-Aldrich) was added to each well with 10 min of incubation. The absorbance of the cell culture at 490 nm was read using a microplate reader (BIO-RAD, Hercules, CA, USA). The experiments were repeated 3 times.

### Statistical analysis

All data obtained from this study were presented as mean ± SD and analyzed using SPSS 21.0 software (SPSS Inc., Chicago, IL) and GraphPad Prism 7.0 software (GraphPad Software, Inc., USA). Differences between groups were analyzed using Student’s *t* test, Chi-square test, and one-way ANOVA followed by Tukey’s or Tamhane’s T2 tests. As each variable approximated the normal distribution according to the Kolmogorov–Smirnov (K-S) normality test results, correlation analysis was performed using Pearson correlation analysis. A receiver operating characteristics curve (ROC) analysis was performed to evaluate the diagnostic value of miR-210. A P value of less than 0.05 indicated statistically significant.

## Results

### Comparison of the demographic and clinical data of patients in each group

The demographic and clinical data of the study population are recorded in Table 2. It was found that there was no significant difference for age, gender, BMI, TG, TC and FINS among different groups (all *P* > 0.05). Compared with the HC group, the levels of FPG, HbA1c, and HOMA-IR were significantly increased in the NDP, NPDR, and PDR groups, and show a gradually increasing trend form the NDP group to the NPDR group to the PDR group. Additionally, the levels of disease course, FPG and HbA1c were significantly higher in both NPDR, and PDR groups than that in NDR group (all *P *< 0.05). Compared with NPDR group, the levels of disease course, FPG, HbA1c and HOMA-IR were significantly increased in PDR group (all *P *< 0.05).

### Expression of miR-210 in the study population

**Fig. 1:**
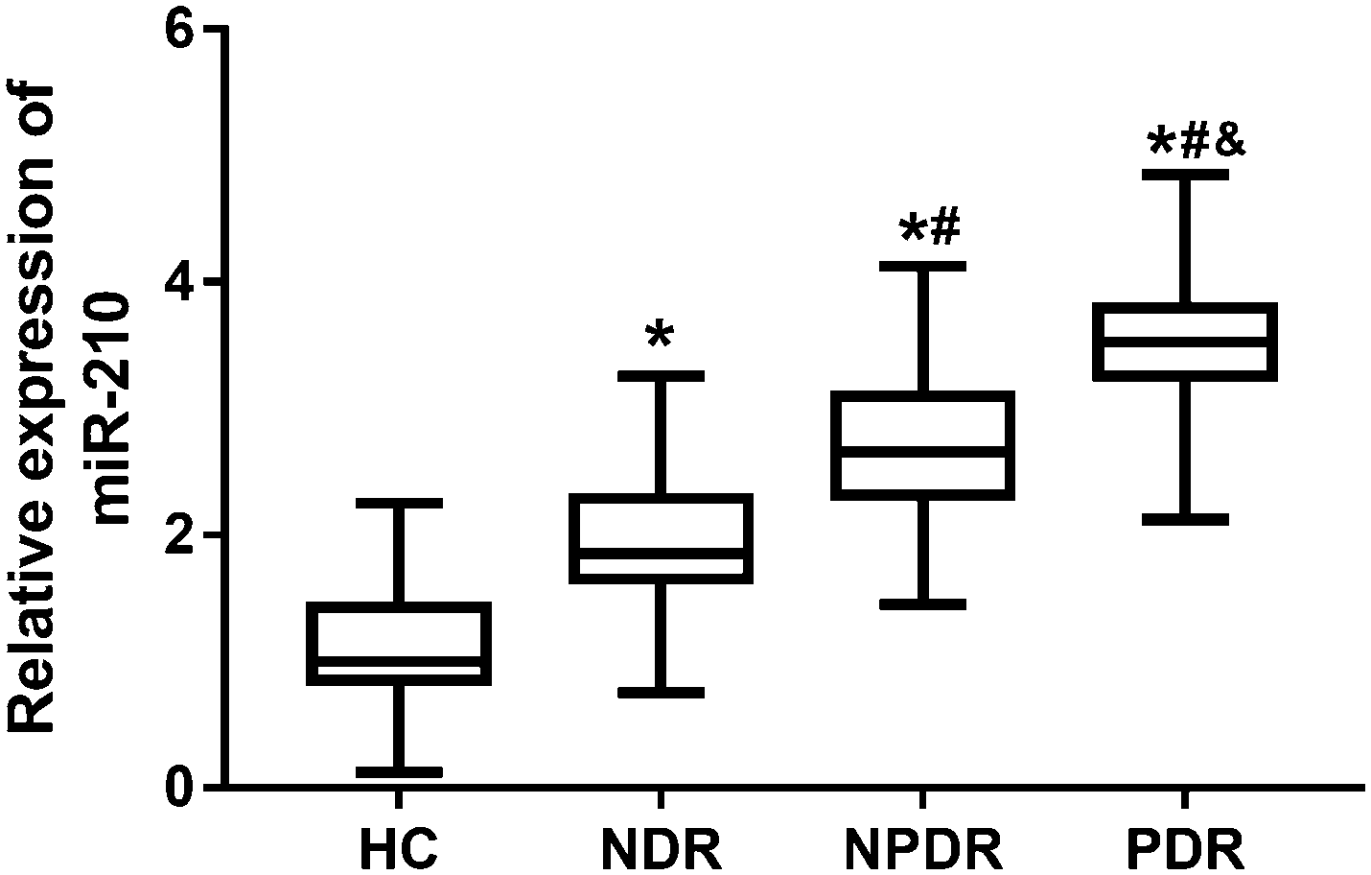
Relative expression of serum miR-210 measured by qRT-PCR in the study population. Compared with HC group, serum expression of miR-210 was upregulated in DM patients, including NDR and DR patients. The expression levels of miR-210 in both the NPDR and PDR groups were significantly increased compared with the NDR group. A significant difference in miR-210 expression was also found between the NPDR and PDR patients. *HC* healthy controls, *NDR* diabetes mellitus patients without diabetic retinopathy, *NPDR* non-proliferative diabetic retinopathy; *PDR* proliferative diabetic retinopathy; *P < 0.05 compared to HC; ^#^*P* < 0.05 compared to NDR; ^&^*P* < 0.05 compared to NPDR. Multiple comparisons were made using ANOVA followed by Tamhane’s T2 tests

According to qRT-PCR, we found that serum expression of miR-210 was upregulated in DM patients, including NDR and DR patients, when compared to the healthy controls (all *P* < 0.05, Fig. 1). The expression levels of miR-210 in both the NPDR and PDR groups were increased compared with the NDR group (both *P* < 0.05). Of note, a significant difference in miR-210 expression was also found between the NPDR and PDR patients, which showed the higher miR-210 expression in the PDR group relative to the NPDR group (*P* < 0.05).

### Correlation between miR-210 and clinical characteristics of DR patients

Pearson correlation analysis results listed in Table 3 show that miR-210 expression was positively correlated with disease course (r = 0.835, *P* < 0.001), FPG (r = 0.798, *P* < 0.001), HbA1C (r = 0.862, *P* < 0.001) and HOMA-IR (r = 0.680, *P* < 0.001). No significant correlation was found between miR-210 and TG, TC, and FINS (all *P* > 0.05).

**Table 3:** Correlation of miR-210 with the clinical characteristics of DR patients

Indicators	MiR-210	
	R	*P*
Disease course	0.835	< 0.001*
FPG	0.798	< 0.001*
TG	0.012	0.203
TC	0.050	0.307
HbA1c	0.862	< 0.001*
FINS	0.033	0.434
HOMA-IR	0.680	< 0.001*

*FPG* fasting plasma glucose, *TG* triacylglycerol, *TC* total cholesterol, *HbA1c* glycosylated hemoglobin, *FINS* fasting insulin, *HOMA-IR* homoeostasis model assessment of insulin resistance

*P < 0.05

### Diagnostic performance of miR-210 in DR patients

**Fig. 2:**
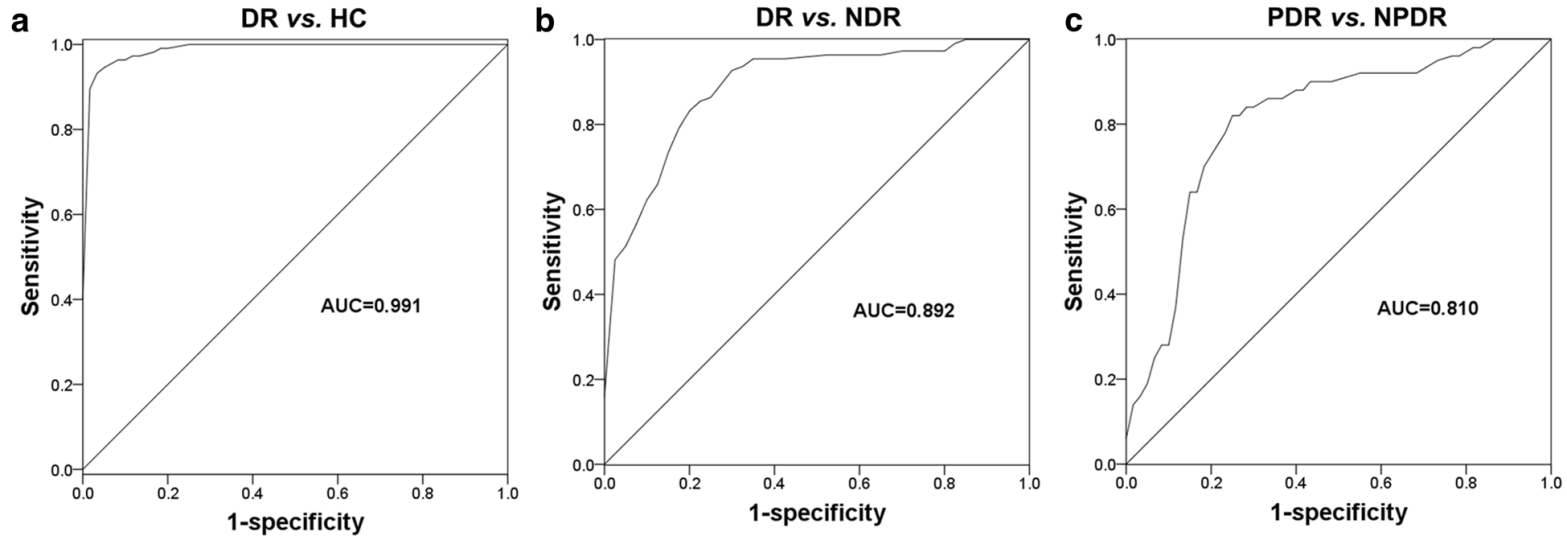
ROCs for DR patients based on the different expressions of serum miR-210. **a** Diagnostic performance of miR-210 in DR patients from healthy controls. The AUC was 0.991, with the sensitivity and specificity were 95.5% and 95.0% at a cutoff value of 1.905. **b** Differentiation between DR patients from NDR patients based on the miR-210 expression. The AUC was 0.892, with a sensitivity of 83.6% and a specificity of 80.0% at a cutoff value of 2.335. **c** miR-210 was used in distinguishing between PDR and NPDR. The AUC was 0.810, with a sensitivity of 82.0% and specificity of 75.0% at a cutoff value of 3.070. *HC* healthy controls, *NDR* diabetes mellitus patients without diabetic retinopathy, *NPDR* non-proliferative diabetic retinopathy, *PDR* proliferative diabetic retinopathy, *AUC* area under the curve

Given the dysregulation of miR-210, this study further evaluated the diagnostic value of miR-210 in the distinguishing patients in different groups using the ROC analysis. As shown in Fig. 2a, serum miR-210 expression could be used to differentiate DR patients from healthy controls with an area under the curve (AUC) of 0.991. The sensitivity and specificity were 95.5% and 95.0%, respectively, at a cutoff value of 1.905. Furthermore, a ROC curve for the differentiation between DR and DM patients without DR showed an AUC of 0.892, a sensitivity of 83.6% and a specificity of 80.0% at a cutoff value of 2.335 (Fig. 2b), indicating that serum miR-210 expression had a relatively high predictive value for DR in DM patients. More important, a ROC curve constructed based on serum miR-210 expression in NPDR and PDR patients yields an AUC of 0.810, with a sensitivity of 82.0% and specificity of 75.0%, and the optimal cutoff value was 3.070 (Fig. 2c), indicating the diagnostic value of miR-210 in distinguishing NPDR and PDR cases.

### Expression of miR-210 in glucose-treated HUVECs

**Fig. 3:**
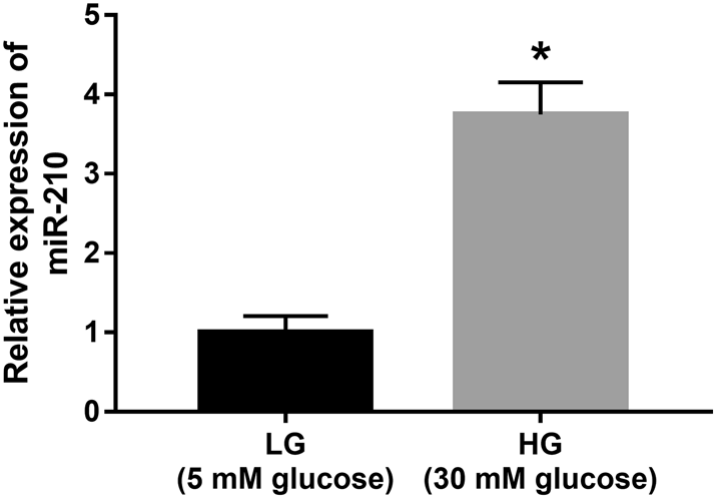
Expression of miR-210 in HUVECs under high-glucose condition. The expression of miR-210 in HUVECs treated with high glucose was significantly increased compared with the cells in the normal control group. *LG* low glucose (5 mM); *HG* high glucose (30 mM); *P < 0.05

The expression of miR-210 in HUVECs treated with high glucose (30 mM) was significantly increased compared with the cells in the normal control group (5 mM glucose) (*P* < 0.05, Fig. 3), which was consistent with the expression tendency of serum miR-210 in DR patients.

### Effect of miR-210 on HUVEC proliferation

**Fig. 4:**
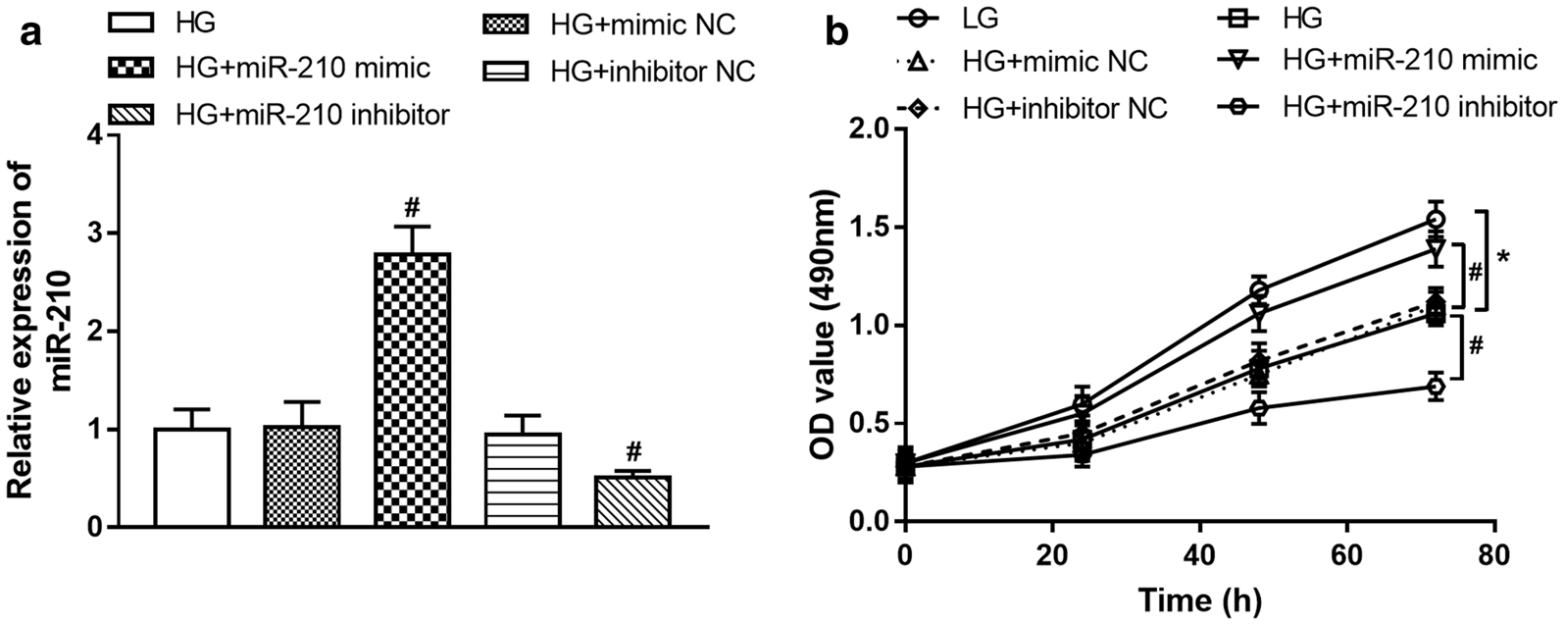
Effect of miR-210 on the proliferation of HUVECs under high-glucose condition. **a** Cell transfection efficiency in HUVECs and miR-210 expression was upregulated by the miR-210 mimic and was downregulated by the miR-210 inhibitor. **b** Regulatory effect of miR-210 on cell proliferation. High glucose led to inhibited HUVEC proliferation, and this effect was ameliorated by the overexpression of miR-210, while it was aggravated by the knockdown of miR-210. *LG* low glucose (5 mM), *HG* high glucose (30 mM); *P < 0.05 compared to LG; ^#^*P* < 0.05 compared to HG. Multiple comparisons were made using ANOVA followed by Tukey’s test

By in vitro manipulation of miR-210, the expression of miR-210 in high glucose-treated HUVECs was upregulated by the miR-210 mimic and was downregulated by the miR-210 inhibitor (all *P* < 0.05, Fig. 4a). The subsequent MTT assay results shown in Fig. 4b showed that the high glucose led to inhibited HUVEC proliferation, and this effect was ameliorated by the overexpression of miR-210, while it was aggravated by the knockdown of miR-210 (all *P* < 0.05).

## Discussion

DM is a common and complex metabolic disorder disease with increasing morbidity worldwide. Long-term hyperglycemia promotes vascular injury, leading to various complications, including DR, diabetic nephropathy, diabetic cataract, diabetic foot, diabetic cardiovascular diseases and diabetic cerebrovascular diseases [18]. A large number of miRNAs have been investigated in DM patients and act as functional roles in DM progression [19, 20]. Furthermore, some miRNAs have also been found to regulate the development of diabetic complications, such as miR-126, miR-23b, and miR-122. Notably, the upregulated expression of miR-210 has been reported in DM patients, and is involved in the progression of DM-associated with coronary artery diseases [21]. The dysregulation of miR-210 has also been suggested to participate in the regulation of diabetes on angiogenesis by regulating endothelial dysfunction in the development of DM-related cardiovascular diseases [12]. In the present study, a total of 150 type 2 DM patients were recruited to explore the expression changes of miR-210. It was noted that the serum miR-210 level was increased in DM patients compared with healthy controls, which was consistent with the previous evidence [21]. Additionally, the enrolled DM patients in the current study were further divided into NDR group, NPDR group and PDR group, and the results indicated that serum miR-210 expression in DR patients was increased compared with DM patients without DR, revealing that miR-210 might also play a role in the development of DR in DM patients.

To understand the mechanism of miR-210 in the development of DM with DR, the relationship between miR-210 and clinical characteristics of DR patients was investigated. Our correlation analysis results revealed that miR-210 expression was positively correlated with disease course, FPG, HbA1c and HOMA-IR. In American Diabetes Association criteria, an FPG of 7.0 mM is a threshold to define the presence of diabetes, which is correlated with the onset of DR [22]. The increased variability of HbA1c is considered to be one of the risk factors of DR [23]. The value of HbA1c needed to be monitored for patients receiving intensive insulin treatment, and is considered as a diagnostic biomarker for DM-associated complications [24]. HOMA-IR is used for the assessment of insulin resistance, the increased HOMA-IR has a strong impact on insulin secretion and the progression of DM [25]. According to the clinical characteristics of DR patients, a close association was detected for serum miR-210 level with disease course, FPG, HbA1c, and HOMA-IR in DM patients. Considering the important role of these markers in reflecting the progression and complication of DM, we deduced that miR-210 might be associated with the development of DR.

Deregulated circulating miRNAs have been proposed as promising diagnostic tools for various human diseases [26]. In this study, increased expression of serum miR-210 was observed in DR patients than that in both DM without DR patients and healthy volunteers. According to the ROC analysis, this study found that using serum miR-210 could distinguish DR patients from healthy individuals, suggesting the diagnostic value of miR-210 for DM. Additionally, considering the dysregulation of miR-210 between NDR and DR patients, we considered that miR-210 might serve as an indicator to predict the onset of DR in DM patients, which was supported by the ROC analysis results. Besides, higher miR-210 expression was found in PDR patients compared with NPDR patients. The subsequent ROC results showed that miR-210 had relative diagnostic accuracy in the distinguishing of PDR from NPDR, indicating that miR-210 could be used to screen PDR cases and might be related to DR severity.

The dysfunction of retinal vascular endothelial cells represents a clinical manifestation of DR. This study used HUVECs to investigate the function of vascular endothelial cells under high-glucose condition. HUVECs have been commonly used in previous studies, including the studies regarding the endothelial cell function in DR [27]. The cell results found that high-glucose treatment led to increased expression of miR-210, which was consistent with the results observed in DM patients. Additionally, the gain and loss function experiments results indicated that overexpression of miR-210 promoted cell proliferation in HUVECs under high-glucose condition. Consistently, the promoting effect of miR-210 on angiogenesis by increasing vascular endothelial cell proliferation has also been reported in several previous studies [13, 14], which supported our present results. Retinal neovascularization is one of the characteristics of DR pathogenesis and involved in the development of PDR [25]. Thus, therapeutic methods involving the regulation of hyperglycemia-induced angiogenesis are developed for the prevention and treatment of DR [28]. A study by Naderi et al. has reported the regulatory role of miR-210 on angiogenesis in DM-related cardiovascular disease [21]. The demethylation of miR-210 could also promote angiogenesis in schwannoma cells under hypoxia [29]. The present results in combination with the regulatory effect of miR-210 on angiogenesis led us to suspect that miR-210 might be involved in the retinal neovascularization in DR pathogenesis. However, the effect of miR-210 on retinal vascular endothelial cell function was not investigated in this study, which may be a limitation of this study. To verify our hypothesis, further studies should focus on the functional role of miR-210 in the development of DM-related retinal endothelial cell injury. Additionally, it is reported that not all miRNAs detected in serum correlate with intravitreal level of the same miRNA in DR patients, but in the present study, only levels of miR-210 in the serum was detected [30]. Further studies are needed to detect the expression changes of miR-210 in the intravitreal of DR patients.

## Conclusion

In conclusion, increased serum miR-210 in DR patients serves as a candidate diagnostic biomarker, which may be used to predict the development of DR in DM patients and associated with DR severity. Besides, miR-210 may be involved in the regulation of vascular endothelial cell proliferation under high-glucose condition, indicating its therapeutic potential in the treatment of diabetic vascular diseases.

## Data Availability

The data that support the findings of this study are available from the corresponding author upon reasonable request.

## Abbreviations

DM: Diabetes mellitus
ROC: Receiver operating characteristics curve
HUVECs: Human umbilical vein endothelial cells
PDR: Proliferative DR
NPDR: Non-proliferative DR
miRNAs: MicroRNAs
FFA: Fundus fluorescence angiography
FPG: Fasting plasma glucose
TG: Triacylglycerol
TC: Total cholesterol
HbA1c: Glycosylated hemoglobin
FINS: Fasting insulin
HOMA-IR: Homoeostasis model assessment of insulin resistance
HUVECs: Human umbilical vein endothelial cells
FBS: Fetal bovine serum
qRT-PCR: Quantitative real-time PCR
ROC: Receiver operating characteristics curve
